# Design and Synthesis of a Quintessential Self-Transmissible IncX1 Plasmid, pX1.0

**DOI:** 10.1371/journal.pone.0019912

**Published:** 2011-05-18

**Authors:** Lars H. Hansen, Mikkel Bentzon-Tilia, Sara Bentzon-Tilia, Anders Norman, Louise Rafty, Søren J. Sørensen

**Affiliations:** 1 Department of Biology, University of Copenhagen, Copenhagen, Denmark; 2 DNA2.0, Menlo Park, California, United States of America; J. Craig Venter Institute, United States of America

## Abstract

DNA exchange in bacteria via conjugative plasmids is believed to be among the most important contributing factors to the rapid evolution- and diversification rates observed in bacterial species. The IncX1 plasmids are particularly interesting in relation to enteric bacteria, and typically carry genetic loads like antibiotic resistance genes and virulence factors. So far, however, a “pure” version of these molecular parasites, without genetic loads, has yet to be isolated from the environment. Here we report the construction of pX1.0, a fully synthesized IncX1 plasmid capable of horizontal transfer between different enteric bacteria. The designed pX1.0 sequence was derived from the consensus gene content of five IncX1 plasmids and three other, more divergent, members of the same phylogenetic group. The pX1.0 plasmid was shown to replicate stably in *E. coli* with a plasmid DNA per total DNA ratio corresponding to approximately 3–9 plasmids per chromosome depending on the growth phase of the host. Through conjugation, pX1.0 was able to self-transfer horizontally into an isogenic strain of *E. coli* as well as into two additional species belonging to the family *Enterobacteriaceae*. Our results demonstrate the immediate applicability of recent advances made within the field of synthetic biology for designing and constructing DNA systems, previously existing only *in silica*.

## Introduction

The term “synthetic biology,” in its present context, covers the creation of specific self-replicating DNA sequences that encode novel functional biological components, or replicas of existing natural systems, based on extensive preexisting knowledge about gene conservation, function and synteny.

The ability to utilize this new technique has, in large part, been facilitated through recent dramatic advances made within the areas of genome sequencing, and now also genome synthesis [1]. Thus, the ground has been laid for a new era in which the design and creation of functional, synthetic self-replicating genomes of increasing size and complexity has been made possible [1]–[10]. In no other field than microbiology are these advances so timely or relevant.

Most bacteria carry extrachromosomal self-replicating elements that aid in adaptation to local conditions in their environment. In enteric bacteria, some of the more interesting of these extrachromsomal elements belong to the relatively unknown IncX1 group of mostly conjugative plasmids. They propagate almost solely within the family *Enterobacteriaceae*, and typically carry genetic loads promoting antibiotic resistance or virulence mostly in the form of cell adhesion factors. The IncX1 plasmids have thus far been reported to confer resistance towards a wide array of antimicrobial compounds, such as the sulfonamides, the ß-lactams, the quinoxaline olaquindox, fluoroquinolones, and chloramphenicol [11]–[13].

The ability of IncX1 plasmids to spread infectiously through bacterial communities puts them, along with bacteriophages and other viruses, into a broader family of molecular parasites that are able to spread between host cells horizontally. The gene content of conjugative plasmids can typically be divided into plasmid-selfish genes that maintain the integrity and propagation of the plasmids themselves, and genes that benefit the host cell in adapting to local conditions (the genetic load). Cryptic plasmids are examples of extrachromosomal parasitic elements that only encode genes essential for plasmid replication and stable maintenance, and are commonly found within the *Enterobacteriaceae* [14]. Some members of the IncP family of relatively large plasmids have been shown to exist without any apparent accessory mobile elements [15]–[17], however, the occurrence of “pure” versions of IncX1 plasmids (i.e. lacking genetic loads) has yet to be elucidated. We therefore embarked on an attempt to recreate such a minimal incX1 plasmid, employing a bottom-up design strategy that would easily accommodate future experimental work and manipulations. Although molecular parasites have been constructed previously from smaller synthesized oligonucleotides [5]–[7], [18], pX1.0 is the first synthetic DNA molecule able to maintain itself in- and spread to several different species of bacteria. Furthermore, it represents the first synthetic DNA construct designed *in silica* as a consensus construct derived from several different members of a discrete phylogenetic group, thus it arguably represents the archetypical IncX1 backbone.

**Figure 1:**
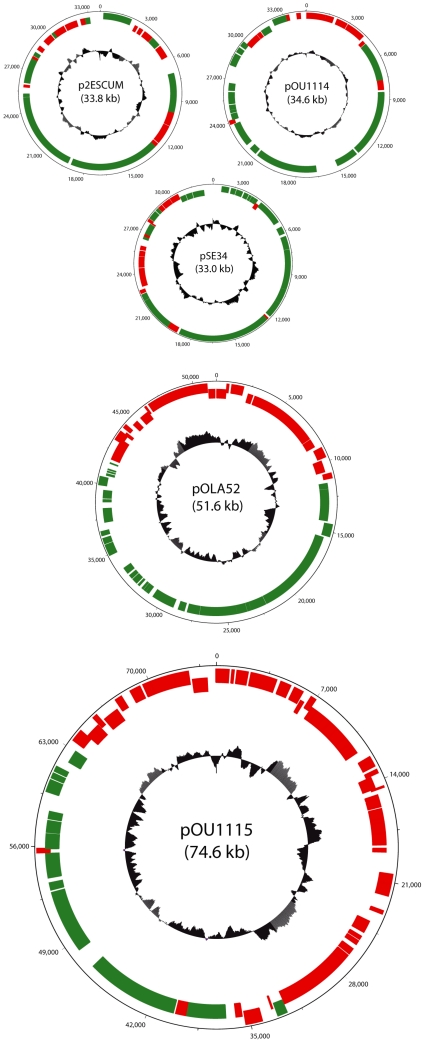
Genetic maps of p2ESCUM, pOU1114, pSE34, pOLA52, and pOU1115. The GC-content is depicted in the central part of the maps. Open reading frames (ORFs) proven to be conserved within the IncX1 plasmids constitute the putative IncX1 backbone and are presented as green boxes, whereas red boxes represent ORFs comprising the genetic load.

The design of the 30.2 kilobase (kb) pX1.0 plasmid was initiated by first selecting the components for a minimal IncX1 sequence. The core sequence is based on a consensus of gene content derived from five publicly available IncX1 plasmid sequences, each consisting of a mostly conserved plasmid backbone, alongside accessory genetic loads of varying sizes (fig. 1). Apart from the consensus IncX1 gene content, pOLA52 carries a genetic load of approximately 22 kb. The load is comprised of the ß-lactamase gene *bla*, an operon encoding a multidrux efflux pump, and a *mrk* operon encoding type III fimbriae [19]. In addition to the putative IncX1 backbone, 4 kb of the 33 kb plasmid pSE34 encodes a series of un-conserved hypothetical proteins. 5 kb of the 35 kb pOU1114 plasmid, and 5 kb of the 34 kb plasmid p2ESCUM encodes a series of un-conserved hypothetical proteins as well. The *Salmonella enterica* serotype Dublin virulence plasmid pOU1115 consists, apart from the consensus IncX1 gene content, of 46 kb of additional DNA including loci encoding K88 fimbriae and proteins involved in proliferation of the bacteria in intestinal and extraintestinal tissues [20].

At the heart of the pX1.0 construct is an archetypical IncX1 replicon which encodes the replication initiation protein π, initiator of plasmid replication through a Cairns type (Θ type) mechanism at three discrete origins of replication (α, ß and γ), along with the Bis protein, required for replication initiation at the ß-origin. Furthermore, the entire conjugational system associated with IncX1 plasmids was included, along with two cognate origins of transfer, to enable horizontal transfer of the construct. The 15.8 kb mob/tra-locus is comprised of 19 ORFs, 16 encoding a type IV secretion system essential for mate-pair formation, and 3 ORF's encoding functions concerned with DNA transfer and post-conjugal replication [19]. To ensure maximum stability, common plasmid stability determinants associated with IncX1 plasmids, such as the ParFG active partitioning system related to the archetypical ParA ATPase/ParB mechanism [21] and the post segregational StbDE toxin/antitoxin killing system were also retained in the base sequence.

**Figure 2:**
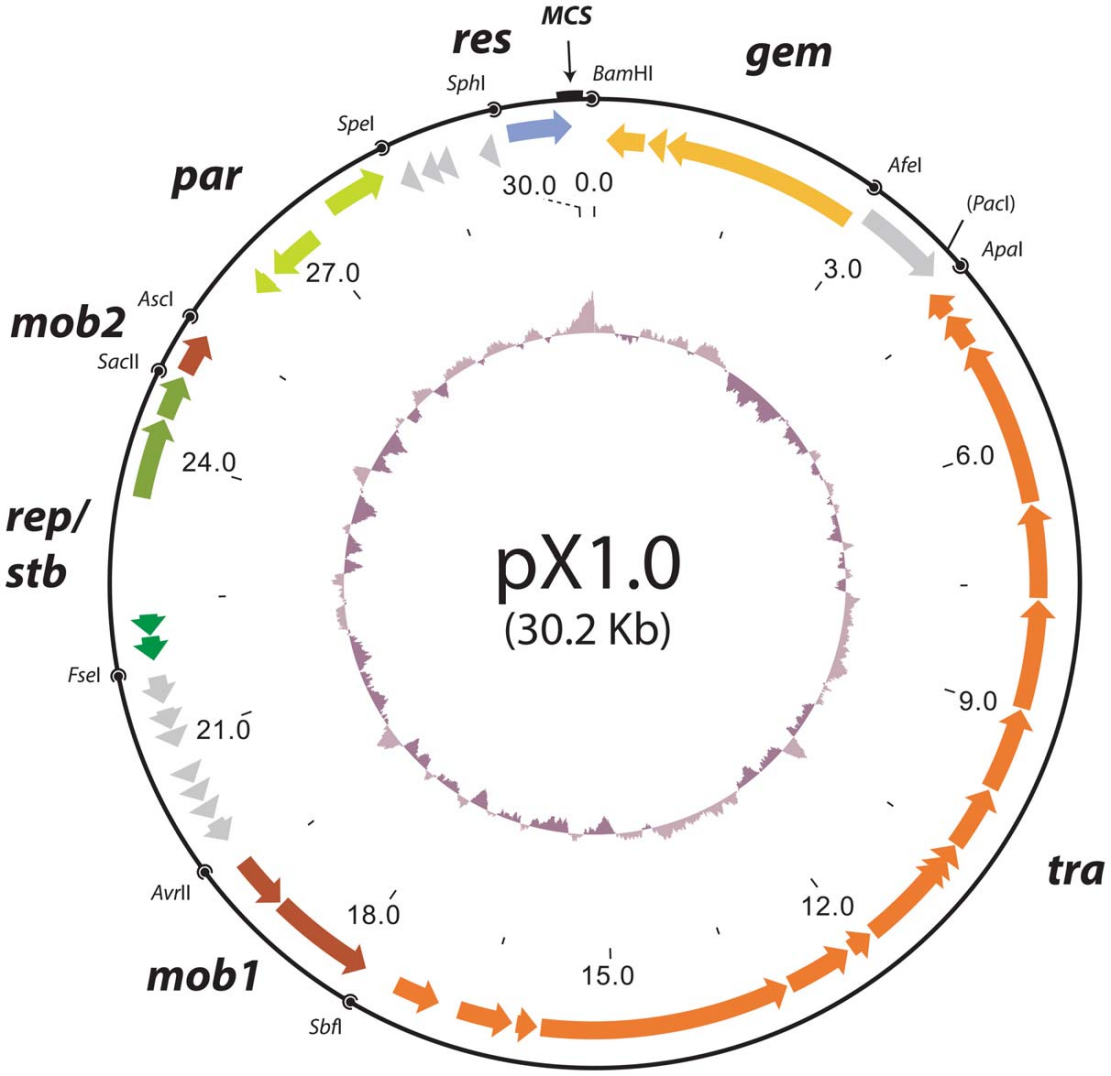
Genetic map of pX1.0. The GC-content is depicted in the central part of the map. Open reading frames are shown as colour coded arrows, indicating their function and transcribed direction. The ORFs are divided into the following modules: gem (gene expression modulation), tra (conjugal transfer), mob1 (mobilization 1), rep/stb (replication initiation/toxin-antitoxin plasmid addiction), mob2 (mobilization 2), par (plasmid partitioning) and res (resistance marker). Unique restriction sites are shown at the module junctions. MCS represents the restriction sites *Pme*I, *Abs*I, *Mre*I, *Kpn*I, *Swa*I, *Not*I and *Sgr*DI.

During the next phase of the design process, emphasis was put on retaining much of the intrinsic modularity of plasmid genomes by surrounding each functional backbone module with unique restriction sites. Restriction sites were carefully chosen based on a normalized frequency index comparing relative restriction-site occurrences in the plasmid database and selected bacterial genomes. Restriction sites chosen to adjoin modules all had maximum digestion efficiencies in the same buffer. Any chosen restriction site re-occurring in the coding regions were removed by silent mutations in accordance with Gene Designer's *E. coli* codon-bias table [22], [23], and restriction sites leaving blunt ends were placed between modules flanked by restriction sites leaving overhangs. Furthermore, a multiple cloning site (MCS) was inserted at a neutral position (29.3 kb–30.2 kb) along with a removable, moderately expressed chloramphenicol acetyltransferase selection marker (*cat*) enabling the application of selective pressure in plasmid transfer- and maintenance experiments. The potential ease, with which future studies on plasmid host range, conjugational efficiency, and other properties of pX1.0 can be carried out, is thus greatly enhanced by the ability to perform discrete manipulations (i.e. removing or replacing modules) with simple molecular techniques such as restriction endonuclease- and ligase reactions. A graphical overview of the entire pX1.0 plasmid sequence is depicted in figure 2.

The synthesis stage involved dividing the entire pX1.0 sequence into three cassettes of approximately 10 kb in length that were then individually synthesized at DNA 2.0, essentially as previously described [10]. Break points were chosen so that neither the central replicon, nor the chloramphenicol resistance marker would be functional prior to a complete assembly of the sequence. Fragments were verified with double-stranded DNA sequencing and joined together at designed restriction sites. Fragment assembly was concluded with a full re-verification (by DNA sequencing) of the plasmid to confirm that no bases differed from the original designed sequence. This was done before any functional experiments were carried out.

During the subsequent verification steps, the construct was demonstrated to replicate with a copy number comparable to most other low-copy number plasmids. In exponentially growing batches of *E. coli* GeneHogs®, pX1.0 replicated at a ratio equivalent to 0.6 plasmid copies per fg total DNA as determined by quantitative PCR (qPCR). During stationary phase the plasmid were found to replicate at a ratio equivalent to 1.8 plasmids per fg total DNA. Assuming that the average molar mass of the 4.7 Mb *E. coli* chromosome is 650 g mol^−1^, pX1.0 is estimated to replicate at a copy number of 3–9 copies per *E. coli* chromosome. The similar IncX1 plasmid R485 has previously been reported to replicate at an average copy number of 3–5 plasmid copies per *E. coli* cell [24] indicating that the pX1.0 replicon operates at levels comparable to its natural equivalents.

To verify functionality of the combined 15,827 bp region containing the tra- and mob modules, conjugation frequencies of pX1.0 from *E. coli* GeneHogs® donors into different members of *Enterobacteriaceae* family, were determined. Conjugation from *E. coli* GeneHogs® into an isogenic strain of *E. coli* was observed in di-parental filtermatings at a mean frequency of 5.5·10^−3^±1.7·10^−3^ (s.d) transconjugants per donor. Compared to the previously reported conjugation frequency of 9.0·10^−2^ transconjugants per donor in the plasmid pOLA52 [25], the observed transfer frequencies of this study suggest that the conjugation apparatus of pX1.0 functions similar to that of pOLA52. Any decrease in conjugal transfer frequencies of pX1.0 compared to pOLA52 may owe to the fact that pOLA52 encodes type III fimbriae, influencing cell proximity, and consequently the frequency of plasmid transfer [25]. In a similar fashion, pX1.0 successfully transferred itself horizontally from *E. coli* GeneHogs® to *Salmonella typhimurium* and *Enterobacter aerogenes* with mean conjugation frequencies of 3.7·10^−4^±2.7·10^−4^ (s.d.) and 4.7·10^−3^±1.8·10^−3^ (s.d.) transconjugants per donor, respectively.

To establish the functionality of the included plasmid maintenance modules, cells harboring the pX1.0 plasmid were propagated in LB medium at 37°C for 50 generations without antibiotic selection. Based on 11 replicas, each representing 96 isolates, the frequency of loss was determined to be 0.004% generation^−1^, as compared to 0.01% generation^−1^ for the naturally occurring IncX1 plasmid pOLA52. A two-tailed, unpaired students *t*-test showed that the observed difference was not statistically significant, with a 95% confidence interval (P = 0.083, *n* = 11). Despite the finding of plasmid free cells, these results show stable inheritance without selective pressure and indicate a parasitic lifestyle of the plasmids.

Incompatibility tests were done as previously described [19], and confirmed that the pX1.0 plasmid was unable to propagate in the presence of the IncX1 plasmid, pOLA52. However, 100% compatibility was observed with the IncX2 plasmid, R6K.

This study presents the first synthetic DNA construct able to replicate within a living organism as well as spread to several different species. We believe that pX1.0 represents the minimal IncX1 backbone, and that this in conjunction with the approach employed in the design process of pX1.0 facilitates further experimental work to a degree that substantiates that pX1.0 becomes a reference plasmid for future studies on the biology of IncX1 plasmids. More importantly, by bringing the hitherto undiscovered quintessential IncX1 plasmid to life, this study illustrates the potential of synthetic biology as a means of reconstructing not only synthetic copies of already existing DNA systems, but also DNA systems existing only *in silica*. It is in this respect it is of utmost importance to emphasize the fact that pX1.0 represents a hypothetical, minimal, non-virulent version of already known incX1 plasmids.

## Materials and Methods

### The design of pX1.0

Based on the consensus gene content of IncX1 plasmids pOLA52, pSE34, pOU1114, pOU1115 and p2ESCUM (GenBank accessions EU370913, EU219533, DQ115387, DQ115388 and CU928149, respectively) a scaffold-sequence representing a putative minimal IncX1 plasmid was designed by removing non-recurring regions of the pOLA52 sequence. Other, more divergent, members of the IncX plasmids (pHI4320, R6K, and pBS512_33) were also used as universal reference points for gene content in the construction of the scaffold. A region of the consensus sequence (19.6 kb–20.7 kb) containing 8 small ORFs were included in the scaffold-sequence because they were found to be universally conserved among the reference plasmids, although their functions remain to be elucidated. Although the active plasmid segregation mechanism (*parFGH*), and the plasmid addiction system (*stbDE*) varied between some of the reference plasmids, the *parFGH* and *stbDE* gene cassettes of pOLA52 were included in the pX1.0 sequence as this required the least amount of sequence manipulations. The essential IncX1 backbone was subsequently modified using the Gene Designer (GD) software from DNA2.0 as follows: All open reading frames (ORFs) and non-coding regions (promoters, origins of replication, and origins of transfer) were reconstructed as discrete DNA elements separated by endonuclease restriction sites. The GD's *E. coli* codon-bias table [22], [23] was used to choose optimal codons for expression in *E. coli* in any situation that required changes to, or addition of, coding regions within the sequence. An artificial chloramphenicol resistance operon, consisting of a *lac*-promoter, a ribosomal binding region containing a Shine-Dalgarno sequence followed by a 7 bp spacer, an *E. coli* optimized version of the chloramphenicol acetyl transferase gene (*cat*), followed by an *rrnB* terminator, was subsequently included. Finally, a multiple cloning site region was included as the last element of the plasmid sequence (see below). A normalized frequency index of all known restriction sites was made, and 20 restriction sites found to be rare in both the plasmid database and in selected bacterial genomes, as well as in non-coding regions of the construct, were selected for further use in the design process. Restriction sites were added in GD as DNA sequence elements, separating the construct into a replication/plasmid addiction module (rep/stb), two mobilization modules (mob1/2), a conjugation module (tra), the segregation module (par), the gene expression modulation module (gem), and the resistance marker module (res). Any restriction sites leading to blunt ends were situated between modules flanked by restriction sites that left overhangs. Any of the 20 restriction site sequences existing naturally in the coding regions were removed by silent mutations. Restriction enzymes with 100% activity in NEB buffer 4 (*Bam*HI, *Afe*I, *Pac*I, *Apa*I, *Asc*I, *Avr*II, *Fse*I, *Sac*II, *Sbf*I, *Spe*I, *Sph*I and *Pme*I) were specifically chosen as module-cutters, so that any module/modules could be excised in a single restriction reaction. The remaining sites (*Abs*I, *Mre*I, *Kpn*I, *Not*I, *Sgr*DI and *Swa*I) were used either in the MCS region, or as restriction site sequences used for adapting fragments to be cloned into the MCS (*Mau*BI and *Xba*I). The full sequence was submitted to GenBank where it was given the accession number HM114226.

### Estimation of the pX1.0 copy number

The pX1.0 copy number was estimated by qPCR. Genomic DNA extractions were made on an overnight (ON) culture of Invitrogen's *E. coli* GeneHogs® harboring the pX1.0 plasmid, and on a similar culture in exponential growth phase. The concentration of total DNA in the two samples was measured using Invitrogen's Qubit™ fluorometer. In order to make a qPCR standard equally complex to the two samples, chromosomal *E. coli* GeneHogs® DNA was added to pX1.0 DNA in the approximate ratio of five plasmids per chromosome. A serial ten-fold dilution of the complex standard was used to make the standard curve. A hundred-fold dilution of sample DNA was used as template in the sample qPCRs. All reactions were carried out using 1 µl of template DNA, and 1 µl of each of the two primers, pX1.0Fw: 5′-CCGTAGCTTGCTCATACATC-3′, and pX1.0Rev: 5′-GTCGTGGTATTCACTCCAGA-3, (10 pmol µl^−1^) in Stratagene's Brilliant® II SYBR®Green qPCR master mix. Finally, the number of plasmids per fg of total DNA in the two samples was calculated and used to estimate the approximate number of plasmids per chromosome.

### Estimating the conjugational abilities of pX1.0

The ability of pX1.0 to conjugate from *E. coli* GeneHogs® to selected enteric bacteria (*E. coli* GeneHogs®::*npt*, *Salmonella typhimurium* DT27 [26], and *Enterobacter aerogenes* DSM30053 (Deutsche Sammlung von Mikroorganismen und Zellkulturen GmbH) was determined by adding 8 µl of donor overnight culture, and 8 µl of recipient overnight culture onto ADVANTEC©'s mixed cellulose/ester membrane filters placed on LB-agar plates. Filter matings were carried out for two hours at 37°C, and the filters were subsequently washed by vortexing the filter in a 0.9% NaCl solution for 3 minutes. A serial ten-fold dilution was made on the wash solution, and droplet assays were carried out on minimal medium containing 0.2% glucose and 5 µg ml^−1^ chloramphenicol for the *E. coli* GeneHogs®+*Salmonella typhimurium* DT27 mating and for the *E. coli* GeneHogs®+*Enterobacter aerogenes* DSM30053 mating. The droplet assay was carried out on LB-agar supplemented with 50 µg kanamycin and 5 µg chloramphenicol ml^−1^ for the *E. coli* GeneHogs®+*E. coli* GeneHogs®::*npt* mating. Donor enumeration was carried out on LB plates containing 5 µg ml^−1^ chloramphenicol. The frequency of conjugation was estimated as transconjugants donor^−1^. Control filter matings with only the donor or recipient strains were carried out to verify the selectivity of the transconjugant plates.

### Verification of the pX1.0 maintenance genes

In order to determine the stability of pX1.0, *E. coli* GeneHogs® cells harboring either pOLA52 or pX1.0 were propagated for 50 generations by periodic dilutions in tubes containing 5 ml LB-broth. Subsequently 96 colonies of each were isolated on LB-agar and replica plated onto LB-agar plates supplemented with either chloramphenicol or ampicillin, depending on which plasmid was tested. 11 replicas were done for each plasmid. A two-tailed, unpaired students *t*-test were used to determine the significance of the differences in the frequencies of plasmid loss observed between the two plasmids.

The incompatibility testing was carried out as described earlier [19].
